# Problem-based learning in clinical bioinformatics education: Does it help to create communities of practice?

**DOI:** 10.1371/journal.pcbi.1006746

**Published:** 2019-06-27

**Authors:** Angela C. Davies, Diane Harris, Amanda Banks-Gatenby, Andy Brass

**Affiliations:** 1 School of Health Sciences, Faculty of Biology Medicine and Health, The University of Manchester, Manchester, United Kingdom; 2 Manchester Institute of Education, University of Manchester, Manchester United Kingdom; University of Toronto, CANADA

## Abstract

We have now reached the genomics era within medicine; genomics is being used to personalise treatment, make diagnoses, prognoses, and predict adverse outcomes resulting from treatment with certain drugs. Genomic data is now abundant in healthcare, and the newly created profession of clinical bioinformaticians are responsible for its analysis. In the United Kingdom, clinical bioinformaticians are trained within a 3-year programme, integrating a work-based placement with a part-time Master’s degree. As this profession is still developing, trainees can feel isolated from their peers whom are located in other hospitals and can find it difficult to gain the mentorship that they require to complete their training. Building strong networks or communities of practice (CoPs) and allowing sharing of knowledge and experiences is one solution to addressing this isolation. Within the Master’s delivered at the University of Manchester, we have integrated group-centred problem-based learning (PBL) using real clinical case studies worked on during each course unit. This approach is combined with a flipped style of teaching providing access to online content in our Virtual Learning Environment before the course. The face-to-face teaching is used to focus on the application of the students’ knowledge to clinical case studies. In this study, we conducted semistructured interviews with 8 students, spanning 3 cohorts of students. We evaluated the effectiveness of this style of teaching and whether it had contributed to the formation of CoPs between our students. Our findings demonstrated that this style of teaching was preferred by our students to a more traditional lecture-based format and that the problem-based learning approach enabled the formation of CoPs within these cohorts. These CoPs are valuable in the development of this new profession and assist with the production of new guidelines and policies that are helping to professionalise this new group of healthcare scientists.

## Introduction

### Genomic revolution in healthcare

Genomics, the study of an individual’s entire genome, is revolutionising healthcare. Expanding far beyond the field of clinical genetics, it is now being used to diagnose disease, make disease prognoses, and predict potential adverse outcomes to treatments and tailor an individual’s treatment [1]. Techniques such as next generation sequencing (NGS) enable rapid sequencing of huge amounts of genetic information from patients, which can be used to personalise medical treatment. Large-scale sequencing projects, such as the 100,000 Genomes Project currently underway in the UK, are helping to develop the capacity for large-scale genomic sequencing of patients within healthcare [2]. As highlighted in two reports by the Association of the British Pharmaceutical Industry (ABPI), in order to fully realise the potential of NGS, healthcare will need to overcome a serious shortage of the bioinformatic skills required to analyse, integrate, and manage the large data sets generated by NGS [3, 4]. In the UK, Health Education England has addressed this by developing the Scientist Training Programmes (STPs), a training programme designed to train and educate scientists working in healthcare. These programmes encompass many areas of genomics, including the Clinical Bioinformatics programme, designed to develop the profession of clinical bioinformatics within the UK National Health Service (NHS) [5]. This programme is 3 years in length, undertaken within a genetics diagnostic laboratory in the NHS. The trainees also study part-time for a Master’s programme delivered at the University of Manchester. With clinical bioinformatics very much in its infancy as a profession, opportunities are abundant to develop professional standards and guidelines. Recent progress has seen the development of a national clinical bioinformatics network under the umbrella organisation of the Association for Clinical Genomic Science (ACGS), which is beginning to develop momentum, including developing guidelines for the profession to work toward [6]. Developing consistent, well-validated NGS workflows and practices for reviewing code and storing genomic data will help to ensure that genomic patient data is analysed consistently and stored in a safe and secure infrastructure. The harmonisation of genomic data analysis, storage, and sharing is essential if healthcare is to reap the benefits of approaches such as whole genome sequencing.

### Isolation in practice

Isolation in practice and lack of exposure to more senior colleagues with relevant experience to learn from can be detrimental to the progression of clinical bioinformaticians during their training, and we have observed this through first-hand experience from our interaction with the trainees on this programme. Bioinformatics is a new profession to healthcare and even more so to the NHS. Clinical bioinformaticians are sparsely distributed across England and Wales and therefore physically isolated from colleagues from whom they may garner expertise and knowledge. Some trainees find it very hard to achieve particular competencies within the workplace due to a deficit of more senior colleagues from which to learn and be mentored by.

Building strong networks or communities of practice (CoPs) (described further below) that allow sharing of knowledge and experiences is one solution to addressing this isolation.

### Communities of practice

CoPs in a professional context can be thought of as networks of individuals engaged in sharing and creating new knowledge [7]. Wenger proposed 3 main dimensions that define a CoP: (i) mutual engagement, which might involve interaction, a sense of belonging, and contribution to and the building of relationships; (ii) joint enterprise, which might involve a collective response by members to their common situation; and (iii) finally, a shared repertoire, which might involve the joint development of approaches, procedures, and activities. Ranmathugala and colleagues (2011) undertook a systematic review to determine the impact of the formation of communities of practice within healthcare. In those studies considered, some were established as management initiatives whilst others arose rather more spontaneously, though in common was the intent to share knowledge and improve practice. Their findings indicated that CoPs either on their own or as part of larger organisational initiatives have a role to play in improving healthcare performance [8].

### Course design and instruction

The structure of the course is presented in Table 1; teaching within the programme is front-loaded, with students undertaking most of their taught modules within the first year. This is to match the needs of the workplace, including a programming and an applied NGS module, as students are quickly expected to become involved in both of these activities within the workplace. We have embedded problem-based learning (PBL) within the course, centred on real clinical case studies, as outlined in Tables 2A and 2B. The modules are taught in a condensed format over 5 days; the week is split between a mix of face-to-face lectures in the mornings and group-centred PBL in the afternoons. The case studies we use are real and are provided by clinical colleagues, thereby ensuring that the experience is authentic to clinical practice. The students work on the case studies within groups carefully selected to include a mix of educational backgrounds, including students that have previous experience of working with bioinformatics tools or of working in a clinical genetics laboratory. A facilitator is assigned to each group of 5–6 students to work with them throughout the practical aspects of the module (usually completed consecutively over 5 days). The groups will give feedback to their facilitator and submit written summaries of their work throughout the week, allowing ample opportunity for formative feedback. Facilitators then convene at the end of each day to discuss any concerns or concepts that may have arisen that need further explanation. This approach enables additional instruction in the form of short tutorials, when needed, preceding the following day’s practical session. At the end of the week, the students present their findings as a group to the course facilitators and clinical colleagues, providing opportunities for reflection and constructive feedback, which is beneficial to the students prior to the completion of their individual assignments.

**Table 1:** Course structure, including unit titles (1 credit is equivalent to 10 notional hours of learning).

Year	Unit Title	Credits
1	Professional Practice and Introduction to Healthcare Sciences	15
1	Clinical Bioinformatics	10
1	Generic Content (Human Physiology)	5
1	Clinical Bioinformatics 2	30
1	Programming	15
1	Applied Clinical Bioinformatics: Applied Next Generation Sequencing	10
2	Advanced Clinical Bioinformatics	15
2	Research Project Part 1	30
2	Research Methods	0
3	Applied Clinical Bioinformatics: Whole Systems Molecular Medicine	10
3	Applied Clinical Bioinformatics: Advanced IT	10
3	Research Project Part 2	30
Total		180

**Abbreviations:** IT, Information Technology.

**Table 2A:** Example structure of morning traditional style lectures in the introduction to clinical bioinformatics module.

Day	Morning (3 hours)	Example Intended Learning Outcomes
1	Introductory lectures on molecular biology, genetics, genomics, and sequencing.	Describe the structure of DNA and the functions of coding and noncoding DNA. Discuss the flow of information from DNA to RNA to protein in the cell.
2	Introductory lectures on bioinformatics, including, primary sequence databases, genome browsers, creating alignments, and assessing homology, genotype, and phenotype ontologies.	Describe appropriate bioinformatics databases capturing information on DNA, RNA, and protein sequences. Explain the theory of sequence analysis and the use of genome analysis tools.
3	Introduction of clinical bioinformatics databases that are useful for assessing the pathogenicity of a genetic variant.	Describe secondary databases in bioinformatics and their use in generating metadata on gene function. Discover resources linking polymorphism to disease processes and discuss and evaluate the resources that are available to the bioinformatician and how these are categorized.
4	Clinical case studies presented by a clinical scientist, using current best practice guidelines to assess all sources of evidence and assign the pathogenicity of a variant.	Describe the biological background to diagnostic genetic testing and clinical genetics and the role of bioinformatics. Describe the partnership of clinical bioinformatics and genetics to other clinical specialisms in the investigation and management of genetic disorders and the contribution to safe and effective patient care.
5	Task 5: Prepare a 15-minute presentation to be given by the group on the analysis on their variant, including a clinical report outlining their assessment of the variant’s pathogenicity.	Apply the knowledge of clinical bioinformatics to address specific clinical problems.

**Table 2B:** Example structure of afternoon group PBL-based activity within the introduction to clinical bioinformatics module.

Day	Afternoon Group Activity (3 hours)	Intended Learning Outcomes	Formative Feedback
1	Investigation of a genetic variant taken from a real clinical genetics case study. Task 1: Research background of disease, gene, protein involved, management, and treatment of the disorder.	Discuss and justify the importance of standards, best practice guidelines, and SOPs and how they are developed, improved, and applied to clinical bioinformatics.	To undertake this task, students are asked to create a SOP; feedback is provided on this SOP and on the information that they have retrieved.
2	Task 2: Locate the gene, correct transcript, and variant. Download homologous sequences, create a sequence alignment, and assess conservation at the position of the variant.	Perform analysis on DNA data and protein sequence data to infer function.	To undertake this task, students are asked to create a SOP; feedback is provided on this SOP and on the information that they have retrieved.
3	Task 3: Analyse clinical bioinformatics databases for presence of the variant and use this evidence to assess the pathogenicity of the variant.	Select and apply appropriate bioinformatic tools and resources from a core subset to typical diagnostic laboratory cases, contextualised to the scope and practice of a clinical genetics’ laboratory.	To undertake this task, students are asked to add to their SOP from day 2; feedback is provided on this SOP and on the information that they have retrieved.
4	Task 4: Bring all lines of evidence together to assess the pathogenicity of the clinical variant.	Communicate complex ideas and arguments in a clear, concise, and effective manner.	Facilitators on hand to field questions from their groups.
5	Task 6: Deliver presentation as a group and answer questions.	Work effectively as an individual or part of a team.	Clinical scientist to ask questions and provide formative feedback on the students’ analyses.

**Abbreviations:** PBL, problem-based learning; SOP, Standard Operating Procedure.

This approach of project- or problem-based learning has not been widely applied or evaluated in bioinformatics education and training but was found to be effective by Emery and Morgan (2017) in a short bioinformatics summer school [9].

**Fig 1:**
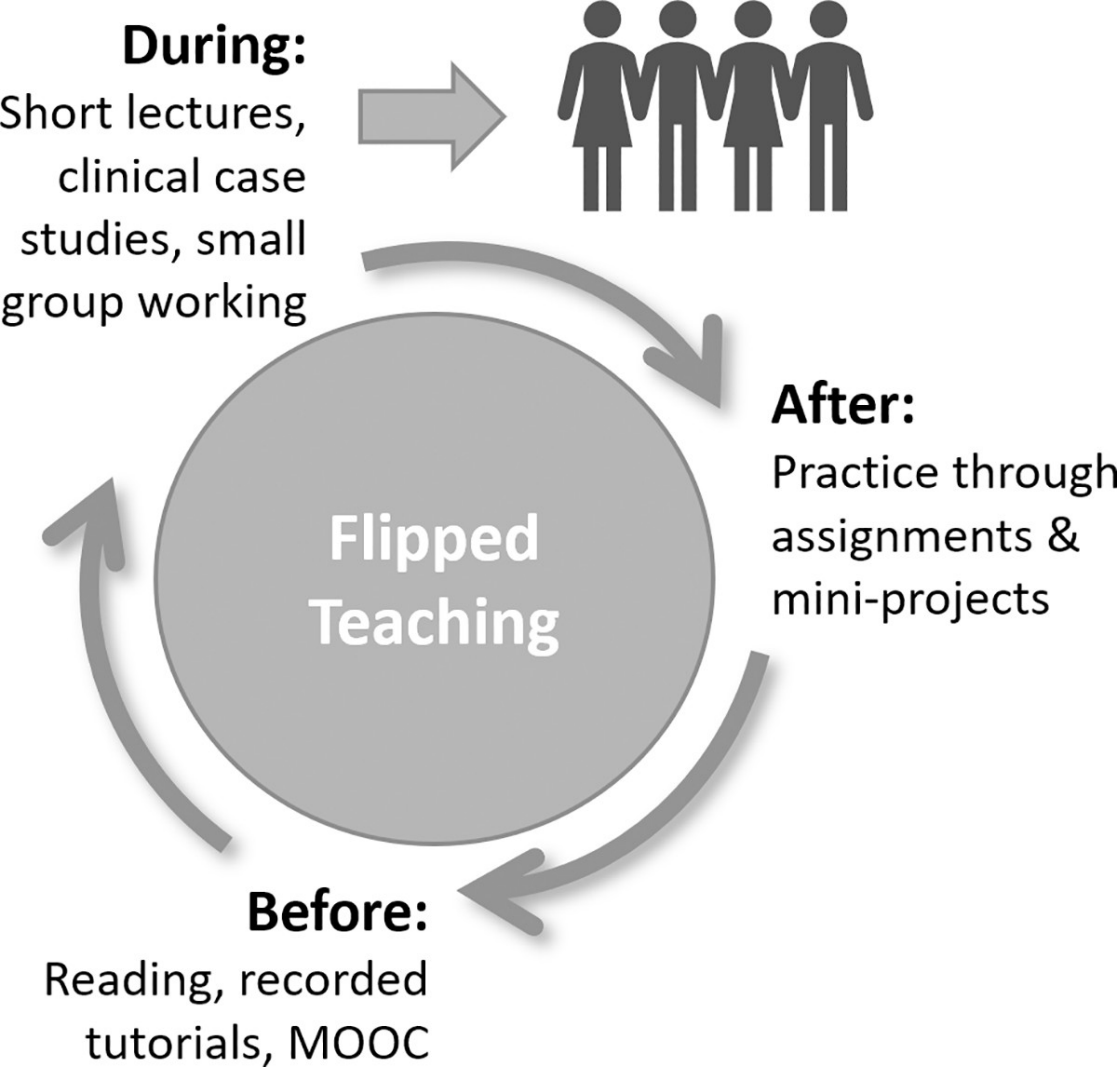
Flipped model of teaching used within the course.

To deliver the curriculum, we have implemented a flipped (or inverted) model of teaching, summarised in Fig 1.

Flipping the classroom in broad terms means that activities that have typically taken place inside the classroom are undertaken outside of the classroom and vice versa. This model of teaching permits access to content, including recorded lectures, tutorials, and Massive Online Open Courses (MOOCs) in advance of face-to-face teaching, allowing contact time to be focused on activities such as PBL. Flipped teaching has been used for some years in higher education; however, it has not been extensively evaluated. Research by Zainuddin and Halili looked at 20 studies on the flipped classroom between 2013–2015, finding that studies focused on a mixed methodology, followed by a quantitative approach with very little focus on qualitative methods [10]. A recent qualitative study by Steen-Utheim and Foldnes investigated the reflections of 12 students who had attained better grades following teaching in a flipped classroom compared to peers receiving traditional lectures within a first-year maths course [11]. Eleven out of 12 students reported that they preferred learning in the flipped classroom; when this was investigated further, the most positive impact on student engagement appeared to be on the affective dimension. For example, students reported a sense of commitment to their peers; they also discussed feeling safe and had a sense of recognition from working in groups.

This style of experiential PBL could be translated and used to educate other healthcare scientists and healthcare professionals. In addition, it might provide a useful approach to base training multidisciplinary healthcare professionals, thus facilitating discussion and exchange of knowledge across professions that otherwise would not regularly come into contact in the workplace.

The knowledge and skills gained within PBL are reinforced through further application to an assessed, individual piece of coursework based on an alternate case study. The notional hours assigned to each element of learning and assessments are indicated below in Table 3.

**Table 3:** Breakdown of study time for the 10-credit introduction to clinical bioinformatics unit.

Learning Component	Hours	Assessment: Percentage of Unit Mark
Online resources, reading, and tutorials	35	-
Face-to-face lectures	12	-
PBL (groups)	18	30%
Individual assignment	25	70%

**Abbreviation:** PBL, problem-based learning.

### Research study aims

Here, we describe a qualitative research study that through semistructured interviews explored the value of a PBL style of teaching with 3 different cohorts of students studying for a Master’s degree in clinical bioinformatics at the University of Manchester. We also investigated whether this style of group-based teaching helped to nurture CoPs, which were then sustained when the students were back in the workplace and often isolated from their peers geographically.

## Methodology

### Research participants

Eight students from across 3 cohorts of students volunteered to be interviewed in October and November 2015. The interviewees were taken from across the 3 cohorts as follows (names are pseudonyms but have been kept as per the gender of the original student): Sarah (first year); Heather, Louise, and Gemma (second year); and Emma, Chloé, Peter, and Ryan (third year). In terms of the cohort size, they were as follows: first year (12), second year (9), and third year (13). Recruitment, interviews, and analysis was undertaken by qualified researchers from the Manchester Institute of Education (MIE) and not by course directors to reduce bias in data collection and analysis.

### Ethics

Ethics approval for this research was obtained from the School of Computer Science, University of Manchester, and consent was obtained from all participants prior to any of the interviews being arranged.

### Interviews

Data was collected in the form of semistructured interviews, which is a well-understood methodology evolving mainly from sociology-based approaches and entails a scheduled prolonged conversation that is structured around a set of predefined questions [12]. This type of interview encourages the interviewee to answer at length and in detail and includes a responsive interview style for which the interviewer may follow the interviewee’s direction [13]. An interview schedule is provided in Table 4; interviews took place in November and December 2015 whilst the students were studying on campus.

**Table 4:** Interview schedule.

Starting question	Elements to draw out with sub questions
1. Experiences of professional practice before starting the course	• Role • Processes • Support from management • Support from/collaboration with colleagues • Contact with those in similar roles • How they felt about the role and themselves within the practice
2. Student view of the structure and content of the course so far	How do they now perceive: • Substantive content, what topics are covered • Teachers • Mode (f-2-f/) • Online and balance between these • Learning materials • Type of activities (reading, preparing presentations etc., individual/group)
3. What aspects of the course so far have stood out	• Positively and negatively • In terms of relevance to their role • Anything that they were uncertain about at that time • Most useful/least useful
4. Relationship with fellow students during the very first taught section of the course	• Level of contact • Experiences of group work
5. Relationship with fellow students now	• Both within course and in practice • Any influence on shared repertoire; do they ‘speak the same language’ more or less, are they ‘on the same page’ more often or no change etc.?
6. Any contact with fellow students between taught sessions	• How often? • By what means? • How is that contact useful to their practice • Do they have a sense that they are all trying to achieve the same things/deal with the same difficulties or not?
7. Any contact with students in other cohorts	• Do they work together either in course or in practice/ locally or virtually? • Do they communicate between sessions? • Do they feel any intrusion from this group • Has it altered the relationship between the first cohort?
8. Student view of which aspects of the course contributed to above (relationship and contact with other students)	Returning to earlier answers, what aspect of the course (as per their perception Q2; content, teachers, activities, etc.) influenced: • Relationship with fellow students and why? • Contact/communication with fellow students and why? • Contact/communication with second cohort and why? • Comparative to University X methods?
9. Predict how course has/will influence future practice	• What roles and ways of working envisaged

The majority of the interviews took place face-to-face at a suitable venue within the university, but there were also two that were conducted by phone. Each interview was planned to last for a maximum of 1 hour, during which time no more than 10 questions would be asked. The student interviews were audio recorded and then formally transcribed, ensuring that due care was taken in anonymising the participant information as well as any comments or notes that could lead to the participants’ identification being deduced by third parties.

### Analysis of interview data

Interviewees have been assigned alternate names to preserve anonymity. The interviews revealed the students’ experiences of and their engagement with the new Clinical Sciences (Bioinformatics) MSc at the University of Manchester and how this shaped their interaction with the other students studying the course. The transcribed texts were analysed using narrative analysis [12].

A narrative is any text or discourse and these naturally occurring language data can be analysed using narrative analysis, comprising a thematic analysis and allowing for a rich, detailed, and complex description of the research data [12]. Initially, 2 of the anonymised interviews were analysed independently by 2 researchers from MIE, and although there was general agreement about the emergent themes, they were discussed at length to ensure that these were relevant the aims of the study. The remaining 6 interviews were then analysed using the identified themes.

### Data availability

All original interview transcript files are encrypted and stored on University of Manchester secure servers available only via institutional login with a password; the full report of the project can be accessed here: http://www.staffnet.manchester.ac.uk/cheril/grants-and-awards/cheril-grant-award-directory/completed-projects/. The report can be found under the 2015/16 subtitle, named under Angela Davies and entitled ‘Flipped teaching report’. Longer term, the report will be archived in the University of Manchester’s library system.

### Results

Ten emergent themes were identified from the data. The 8 substantial interviews (over 500,000 words in total) were coded according to the themes outlined in Table 5.

**Table 5:** Ten emergent themes identified from interview transcripts.

Theme	Additional Comments
Communities of practice	shared repertoire of new practice in bioinformatics.
New career	specialism has no history and so depends on negotiation with potential employers.
Isolation in practice	students talk about being ‘the only one’ in the hospital.
Accountability	the practice is not established.
Teaching/learning	group work, traditional lectures, flipped teaching.
Ownership of the course	student feedback has influenced subsequent presentations of the course.
Joint enterprise	nothing decided by the institutions/professional body so ‘joint enterprise’ is the decision, responsibility of the group.
Talk about group and dynamics	not during studies but social aspects and holidays.

The following questions were addressed in the analysis of the research findings: to what extent do the students experience isolation in practice, to what extent do CoPs exist within the clinical bioinformatics cohorts, and whether the course design helped to nurture and create CoPs.

### To what extent do the students experience isolation in practice?

Isolation within clinical bioinformatics can be problematic, leading to silos where trainees may not get the required support or mentorship to complete their professional competencies. Responses indicated that students did experience isolation within their practice but that their peer group acted as a rich source of support to the development of their knowledge and skills.

Heather was asked if she received any support from senior colleagues:

Not really no…I obviously had my training officer and, another girl who’s in the year above me who’s on the same course, but I think it’s quite a widespread problem of there's not very many specifics on what our role will be in the NHS…I think it’s a bit kind of do what is appropriate at the time to help people out, things like that, and learn on the way. (Heather)

Gemma described how she values the support she receives from the other students in her cohort:

As a cohort, we're quite good friends, we keep in contact constantly…I think that group work really helps with that so if I've got any problems or any questions even though I am isolated within my department, I've always got the other people on my cohort to refer back to. (Gemma)

### To what extent do CoPs exist within the clinical bioinformatics cohort?

Although clinical bioinformatics is a shared endeavour for the postgraduates, their responses suggest that their interactions within their year group versus across different year groups does vary.

#### Within each year group

Students come to the university to study in condensed block release from the workplace between 3 to 4 weeks per year; the rest of the time is spent training in the workplace. To keep in touch in between periods of study, each of the year groups has established a means of communicating, varying from email, to WhatsApp, and to a web-based forum. These methods of communication were not just used to discuss their academic work but also work-related issues:

So if we have issues with anything from coursework to lab work or like, for example, if I wanted to do a survey with the students or find out all their opinions on something, then I have no problem doing that I can just email them or text them. (Heather)

The first few weeks of the training programme can be a stressful time; students need to adjust to a new working environment (often relocating), adjust to recording of work-based competencies, and spend 3 weeks studying away at the university. Students discussed the networks they have formed and that they provided a strong sense of support during a stressful first few months as trainees:

There was a lot of emails going back and forth going, ‘what are we doing? Help!’ …everyone was helping each other out, it kind of felt like we could just push through. (Chloe)

In addition to this remote support through the established networks, students also discussed supporting one another within the problem-based group work undertaken in the face-to-face teaching; Peter discussed how the students’ different backgrounds were used to good effect to support one another:

You get people who are good and are willing to do the explaining but there's certainly been loads, you know, loads of stuff that I have just been not very familiar with but, you know, people have done it, covered it with their PhDs or their previous jobs or whatever and have been able to explain that to other members. (Peter)

#### Across each of the 3-year groups

Some students were lucky enough to have trainees from other cohorts within their laboratory that they could go to for advice. However, for others, there was no real communication with other year groups. Students were in favour of increasing these lines of communication in the future, particularly encouraging it within the academic setting.

Yeah so it would be really beneficial I think if there was some way of getting us to do stuff together at some point, you know, maybe towards the end of the first year, you know, meeting with the second years, even if it is not a ‘third years meet first years’ but at least know the year below. (Chloe)

#### On placement

Students also commented on the value of web-based fora such as Google groups for communicating and sharing resources and information from the university whilst away on their training placements; they also seemed to value the alignment of the academic work closely with their training placements.

### To what extent has the course design helped to create and nurture CoPs?

#### Use of group work to nurture CoPs

For each module, the groups are changed to add variety and ensure that the same students are not always working together, and this seemed to be appreciated by the students:

So they have made it so that we’re not always in the same group which is really good because it means you don't stuck stick to your friend Betty, etc. (Chloe)

Students discussed the advantages of group-based work, being able to learn from one another and share expertise, particularly due to the diverse backgrounds of the students. Drawbacks were discussed particularly in relation to very experienced group members potentially dominating certain tasks within the group. This point was expanded further to discuss that this in turn may mean that some group members would get no exposure to completion of that particular aspect of the task, thus negatively impacting their learning experience. Heather discusses the positive impact that learning in groups has had on her studies, whilst Chloe mentions some of the more negative aspects:

One of the girls had quite a lot of experience of looking at the files that we were trying to extract the data from whereas I'd had experience in outputting a file using code so I was able to bring that experience, she was able to show me what it was that they wanted from the document. (Heather)
It got done too quickly by the person that really knew what they were doing and the rest were just kind of a bit tense. (Chloe)

When Ryan was asked if he thought the communication within the cohort would have still existed without the integration of the group work, he responded:

Because the groups kept changing, you’d go and spend some kind of time in very, very small groups together, and eventually, you know, you get to know each other whereas I think if you were just sitting in lectures you might talk to people sat next to. (Ryan)

### Views on flipped teaching versus traditional lectures

We try to flip the classroom as much as possible by providing as much of the content as possible via Manchester’s virtual learning environment. We have also now embedded the Massive Online Open Course (MOOC) that we have developed in clinical bioinformatics into the flipped approach (https://www.futurelearn.com/courses/bioinformatics), thus enabling face-to-face teaching to focus on the application of the students’ knowledge to clinical case studies. This model varied significantly from the more traditional style used for some of the teaching at University X, from which some of the teaching for this course is delivered. Emma found the flipped approach helpful but sometimes had problems in trying to fit the reading into the time available prior to the sessions.

sometimes there's, we've been given a few papers to read and things but then, you know, you start reading them and you think ‘oh I don’t understand this’ or you start reading them but when you've got time, and it goes well, so I was reading some on the train last night but I was just like ‘I just I just can’t, I can’t follow this paper’, you know, sometimes they start I think a bit too hard. (Emma)

Gemma found this approach useful in preparing her for the group-based work.

Gemma: I think they do really well preparing us for that sort of thing most of the time.

Interviewer: Okay but it’s not that you're not kind of told too much? So do you still feel like you're learning from the group task as well?

Gemma: Yeah exactly and then if you get really stuck, erm, there's always multiple people that help you and make you feel a bit better about it, so yeah, I think they are good at doing that.

Louise found the preparation materials useful as they helped her to understand the underlying algorithms of the software:

It’s not that they kind of give you a task and say ‘go away and do it’. You've kind of had that teaching of what's actually going on underneath and then you do the underneath and then they show you the top, erm, and it’s logical when you think about it. (Louise)

Although Louise appreciated the value of the flipped teaching because it had helped her to understand ‘at a kind of deeper level’ what the computer program was actually doing because she’d done it by hand first, on occasions, she did also find this approach frustrating:

I sometimes think the lectures and the tasks are a bit backwards in terms, just in terms of like we’d have an aim for that kind of group work session and then the next morning they'd say ‘oh so you spent a really long time doing this and now we're going to show you a really quick way to do it’ and it’s kind of it’s sometimes a bit frustrating because you've spent 3 hours working on something that, once you've had that second lecture, takes you ten minutes. (Louise)

## Discussion

Within this study, we have explored the value of a PBL style of teaching with 3 different cohorts of students studying for a Master’s degree in clinical bioinformatics at the University of Manchester. We also investigated whether this style of group-based teaching helped to nurture CoPs and examined whether these were sustained when the students returned to the workplace.

The course design had focused on a flipped approach, with group-centred PBL integrated throughout. Most students enjoyed working in groups and developing connections with peers they might not otherwise have developed and enjoyed being able to learn from the expertise and experience of their colleagues. In some instances, the level of prestudy was cited as being problematic, with amounts of papers and materials being described as overwhelming.

Most of the students’ backgrounds are heavily academic, generally tending to already have completed doctoral- and postdoctoral-level education. Their undergraduate educational experience is largely grounded in a more traditional lecture-based approach, though many will be used to self-directed study gained from their postgraduate experiences. We acknowledge that perhaps allowing dedicated time to discuss the advantages of the educational approach that we have chosen, particularly in relation to them developing in their roles as healthcare practitioners and the codevelopment of CoPs, might be beneficial. This could include some explanation of the design of the course to adhere much more to level 7 descriptors; these are the descriptors set by the Quality Assurance Agency for Higher Education (QAA) in the UK for Master’s level education. The expectation is that at this level, learning will be much more student-led and students will focus on the application of knowledge and the evaluation of tools and methods to case-based problems and that they would be able to critically appraise and formulate appropriate solutions [14].

Some students did describe the pitfalls of a more dominant group member commandeering a particular aspect of the group work such that other students got less exposure to it. This aspect should be given some consideration and reinforces our preference for the course lead to select group members rather than self-organisation.

In order for a CoP to exist, the other elements outlined by Wenger included a joint enterprise (i.e., working together toward a common goal) and a shared repertoire (e.g., common resources and terminologies that participants use to negotiate meaning and facilitate learning within the group and ways of addressing recurring problems). Both of these aspects were described by the students in reference to their participation in the group work on campus but also the sharing of resources particularly whilst on placement to facilitate their development and fulfil professional competencies that they are expected to achieve to complete their training. Within each of the 3 cohorts explored within this study, there was evidence that the group-based work on campus had helped to enable the development of 3 discreet CoPs. These were then sustained by on-going interaction via social apps whilst away from campus on placement.

During the interviews, there was no evidence for the formation of CoPs that transcended the cohorts, though many of the students spoke of the potential benefits of cross-cohort networks and would welcome this, particularly for the on-going development of the profession and the creation of professional guidelines and standards. This is clearly an area that the profession as a whole should be mindful of in its evolution over the coming years, particularly if it is to establish itself as a profession with equal standing and representation to other well-established clinical science roles. We know already that many of our students have joined the newly formed UK-wide clinical bioinformatics network, though we will be interested to consider the longevity of each of the individual CoPs and whether they have been sustained past the completion of the course.

## Conclusions

### What’s worked?

Each CoP that was referred to here was established organically by the students and maintained using social media or other social networking tools such as WhatsApp or Google groups. This was driven by the students using the medium of their choice; this suggests that trying to force this interaction by the development of a discussion space within a virtual learning environment, unless linked to unit assessment, is unlikely to be effective.

Evidence from within this study suggests that each cohort has developed its own individual CoP and that the integration of group-based work in class had enabled the development of each CoP.

### How could we encourage the development of these CoPs further?

The development of a CoP that transcends the cohorts would be beneficial in developing this new profession and help to give it equal standing to other clinical science roles. By increasing the size of the community, clinical bioinformaticians would have a stronger voice to address the issues that they face professionally and would also enable the development of national guidelines and policy. The formation of a professional body to represent the profession would then ensure equal representation of clinical bioinformaticians compared to other clinical scientists working in the area of genomics represented by the ACGS.

Since this study was undertaken, a national clinical bioinformatics network has been developed under the auspices of the ACGS, which meets biannually. However, there are other activities that could be undertaken within the course to augment the development of this national network, including cross-cohort symposia encouraging the presentation of posters and talks, peer–peer assessment across year groups, social events beyond the core curriculum, and peer mentoring.

It is our aim within the course to work in partnership with the newly created national network to further facilitate its development. This might be achieved through the creation of jointly run meetings and initiatives that link the work-based training and educational environments together.

### Translation of this approach

Since this study was undertaken, we have implemented this style of teaching to teach multidisciplinary groups, including mixed groups of trainee genetic counsellors, genomic clinical scientists, and clinical bioinformaticians. We plan to evaluate the success of this teaching style with these broader groups of students and see whether they have formed broader CoPs in a follow-up qualitative research study. The results of this study will be interesting as, if successful, it would help to develop professional networks between colleagues likely to be working in multidisciplinary teams together within the workplace. We have also begun to disseminate this research internally and hope that some courses that are more traditionally lecture-based will consider whether some of their teaching may be delivered in this style.

## References

[1] Davies SC. Annual Report of the Chief Medical Officer 2016, Generation Genome, London: Department of Health 2017 [cited 2017 28 Aug]. Available from: https://www.gov.uk/government/uploads/system/uploads/attachment_data/file/631043/CMO_annual_report_generation_genome.pdf.

[2] 100,000 genomes project [cited 2017 28th Aug]. Available from: https://www.genomicsengland.co.uk/the-100000-genomes-project/.

[3] ABPI. Skills needed for biomedical research 2008 [cited 2017 28 Aug]. Available from: http://www.abpi.org.uk/about-us/resources/publications-library/skills-biomedical-research.

[4] ABPI. Bridging the Skills gap in the biopharmaceutical industry 2015 [cited 2017 28 Aug]. Available from: http://www.abpi.org.uk/our-work/library/industry/Documents/Skills_Gap_Industry.pdf.

[5] Department of Health. Modernising Scientific Careers: The UK Way Forward 2010 [cited 2017 28 Aug]. Available from: https://www.gov.uk/government/uploads/system/uploads/attachment_data/file/138326/dh_113990.pdf.

[6] Association for Clinical Genomic Science [cited 2017 28 Aug]. Available from: http://www.acgs.uk.com/.

[7] Wenger E. Communities of practice: learning as a social system. The Systems Thinker1998.

[8] Ranmuthugala G, Plumb JJ, Cunningham FC, Georgiou A, Westbrook JI, Braithwaite J. How and why are communities of practice established in the healthcare sector? A systematic review of the literature. BMC Health Services Research. 2011;11(1):273. 21999305 10.1186/1472-6963-11-273 PMC3219728

[9] Emery LR, Morgan SL. The application of project-based learning in bioinformatics training. PLoS Comp Biol. 2017;13(8):e1005620. 10.1371/journal.pcbi.1005620 PMC5560525 28817584

[10] Zainuddin Z, Halili SH. Flipped Classroom Research and Trends from Different Fields of Study. 2016 2016;17(3).

[11] Steen-Utheim AT, Foldnes N. A qualitative investigation of student engagement in a flipped classroom. Teaching in Higher Education. 2018;23(3):307–24.

[12] Creswell JW. Qualitative enquiry and research design: Choosing among five approaches.: Thousand Oaks, CA: Sage Publications; 2007.

[13] Rubin HJ, & Rubin I. S. Qualitative interviewing: The art of hearing data.: Thousand Oaks, CA: Sage Publications; 2011.

[14] UK Quality code for HE: QAA; 2014. Available from: http://www.qaa.ac.uk/quality-code/the-existing-uk-quality-code/part-a-setting-and-maintaining-academic-standards.

